# A General Class of Derivative Free Optimal Root Finding Methods Based on Rational Interpolation

**DOI:** 10.1155/2015/934260

**Published:** 2015-01-28

**Authors:** Fiza Zafar, Nusrat Yasmin, Saima Akram, Moin-ud-Din Junjua

**Affiliations:** Centre for Advanced Studies in Pure and Applied Mathematics, Bahauddin Zakariya University, Multan 60800, Pakistan

## Abstract

We construct a new general class of derivative free *n*-point iterative methods of optimal order of convergence 2^*n*−1^ using rational interpolant. The special cases of this class are obtained. These methods do not need Newton's iterate in the first step of their iterative schemes. Numerical computations are presented to show that the new methods are efficient and can be seen as better alternates.

## 1. Introduction

The problem of root finding has been addressed extensively in the last few decades. In 1685, the first scheme to find the roots of nonlinear equations was published by John Wallis. Its simplified description was published in 1690 by Joseph Raphson and was called Newton-Raphson method. In 1740, Thomas Simpson was the first to introduce Newton's method as an iterative method for solving nonlinear equations. The method is quadratically convergent but it may not converge to real root if the initial guess does not lie in the vicinity of root and *f*′(*x*) is zero in the neighborhood of the real root. This method is a without-memory method. Later, many derivative free methods were defined, for example, Secant's and Steffensen's methods. However, most of the derivative free iterative methods are with-memory methods; that is, they require old and new information to calculate the next approximation. Inspite of the drawbacks of Newton's method, many multipoint methods for finding simple root of nonlinear equations have been developed in the recent past using Newton's method as the first step. However, many higher order convergent derivative free iterative methods have also appeared most recently by taking Steffensen type methods at the first step. A large number of optimal higher order convergent iterative methods have been investigated recently up to order sixteen [1–3]. These methods used different interpolating techniques for approximating the first derivative.

In the era of 1960–1965, many authors used rational function approximation for finding the root of nonlinear equation, for example, Tornheim [4], Jarratt, and Nudds [5].

In 1967, Jarratt [6] effectively used rational interpolation of the form

(1) $$\begin{array}{c} y=\frac{x-a}{bx^{2}+cx+d}, \end{array}$$

to approximate *f*(*x*) for constructing a with-memory scheme involving first derivative. The order of the scheme was 2.732 and its efficiency was 0.2182.

In 1987, Cuyt and Wuytack [7] described a with-memory iterative method involving first derivative based on rational interpolation and they also discussed two special cases of their scheme having order 1.84 with efficiency 0.1324 and order 2.41 with efficiency 0.1910.

In 1990, Field [8] used rational function to approximate the root of a nonlinear equation as follows:

(2) $$\begin{array}{c} x_{i+1}=x_{i}+d_{i}, \end{array}$$

where *d*_*i*_, the correction in each iterate, is the root of the numerator of the Pade approximant to the Taylor series:

(3) $$\begin{array}{c} f\left(x\right)=\overset{\infty }{\underset{j=0}{\sum }}\frac{f^{\left(j\right)}\left(x_{i}\right)}{j!}{\left(x-x_{i}\right)}^{j}. \end{array}$$

He proved that *x*_*i*_ converges to the root *ξ* with order *m* + *n* + 1, where *m* and *n* are degrees of the denominator and numerator of the Pade approximant.

In 1974, Kung and Traub [9] conjectured that a multipoint iterative scheme without memory for finding simple root of nonlinear equations requiring *n* functional evaluations for one complete cycle can have maximum order of convergence 2^*n*−1^ with maximal efficiency index 2^(*n*−1)/*n*^. Multipoint methods with this property are usually called optimal methods. Several researchers [1–3, 10] developed optimal multipoint iterative methods based on this hypothesis.

In 2011, Soleymani and Sharifi [10] developed a four-step without-memory fourteenth order convergent iterative method involving first derivative having an efficiency index of 1.6952. The first derivative at the fourth step is approximated using rational interpolation as follows:

(4) wnϕ8xn,yn,znxn+1 =wn−1+b4wn−xn2f′xn+b3wn−xn2+b4wn−xnfwn,

where *ϕ*_8_ is an optimal eighth order convergent method and the rational interpolant is given as:

(5) $$\begin{array}{c} m\left(t\right)=\frac{b+b_{2}\left(t-x\right)+b_{3}{\left(t-x\right)}^{2}}{1+b_{4}\left(t-x\right)}. \end{array}$$

In 2012, Soleymani et al. [3] developed a three-step derivative free eighth order method using rational interpolation as follows:

(6) zn=ϕ4xn,yn,wn,xn+1 =zn−1+a3zn−xn2a1−a0a3+2a2zn−xn+a2a3zn−xn2fzn,

where *ϕ*_4_ is any two-step fourth order convergent derivative free iterative method. They used the same rational interpolant as given in (5). The constants are determined using the interpolating conditions.

In 2012, Soleymani et al. [2] added his contribution by developing a sixteenth order four-point scheme using Pade approximation. The scheme required four evaluations of functions and one evaluation of first derivative and achieved optimal order of sixteen and and an efficiency index 1.741. The scheme was of the form

(7) wn=ϕ8xn,yn,zn,xn+1=wn−1+b5wn−xn2fwn   ×f′xn+2b3wn−xn+3b4+b3b5wn−xn2     +2b4b5wn−xn3−1,

where *ϕ*_8_ is eighth order optimal method. They used the rational interpolant of the following form:

(8) $$\begin{array}{c} p\left(t\right)=\frac{b_{1}+b_{2}\left(t-x\right)+b_{3}{\left(t-x\right)}^{2}+b_{4}{\left(t-x\right)}^{3}}{1+b_{5}\left(t-x\right)}. \end{array}$$

Recently in 2013, Sharma et al. [1] developed a three-step eighth order method and its extension to four-step sixteenth order method using rational interpolation. In the scheme, the first three steps are any arbitrary eight order convergent method. The fourth step is the root of the numerator of the method, which is given as

(9) tn=ϕ8xn,zn,wn,xn+1 =xn−P1fzn,wn+Q1fxn,wn+Rftn,wnP1L+Q1M+RNfxn,

where,

(10) L=fwnfxn,zn−fznfxn,wnwn−zn,M=fwnf′xn−fxnfxn,wnwn−xn,N=fwnfxn,tn−ftnfxn,wnwn−tn,P1=xn−tnfxnftn,Q1=tn−znftnfzn,R=zn−xnfznfxn,

and rational polynomial of the following form:

(11) $$\begin{array}{c} p_{4}\left(x\right)=\frac{\left(x-x_{i}\right)+\lambda }{\mu {\left(x-x_{i}\right)}^{3}+\nu {\left(x-x_{i}\right)}^{2}+\xi \left(x-x_{i}\right)+\eta }. \end{array}$$

The efficiency index of the above sixteenth order method is 1.741. The method involves one derivative evaluation.

In this paper, we present a general class of derivative free *n*-point iterative method which satisfies Kung and Tarub's Hypothesis [9]. Proposed schemes require *n* functional evaluations to acquire the convergence order 2^*n*−1^ and efficiency index can have 2^(*n*−1)/*n*^. The contents of the paper are summarized as follows. In Section 2, we present a general class of *n*-point iterative scheme and its special cases with second, fourth, eighth, and sixteenth order convergence.  Section 3 consists of the convergence analysis of the iterative methods discussed in Section 2. In the last section of the paper, we give concluding remarks and some numerical results to show the effectiveness of the proposed methods.

## 2. Higher Order Derivative Free Optimal Methods

In this section, we give a general class of *n*-point iterative method involving *n* functional evaluations having order of convergence 2^*n*−1^. Thus, the scheme is optimal in the sense of the conjecture of Kung and Traub [9].

Consider a rational polynomial of degree *n* − 1 as follows:

(12) $$\begin{array}{c} r_{n-1}\left(t\right)=\frac{p_{1}\left(t\right)}{q_{n-2}\left(t\right)}, \end{array}$$

where,

(13) $$\begin{array}{c} p_{1}\left(t\right)=a_{0}+a_{1}\left(t-x\right),\\ q_{n-2}\left(t\right)=1+b_{1}\left(t-x\right)+\cdots +b_{n-2}{\left(t-x\right)}^{n-2},\;n\geq 2,\\ q_{0}\equiv 1. \end{array}$$

We approximate *f*(*x*) by rational function given by (12) to construct a general class of *n*-point iterative scheme. Then, the root of nonlinear equation *f*(*x*) = 0 is the root of the numerator of the rational interpolant of degree *n* − 1 for the *n*-point method. The unknowns *a*_0_, *a*_1_, *b*_1_,…, *b*_*n*−1_ are determined by the following interpolating conditions:

(14) $$\begin{array}{c} r_{n-1}\left(x\right)=f\left(x\right),\\ r_{n-1}\left(w_{k}\right)=f\left(w_{k}\right),\;\;k=1,\ldots ,n-1,\;\;n\geq 2. \end{array}$$

Then, the general *n*-point iterative method is given by

(15) w1=x+βfx,  ⋮wn=x−a0a1, n≥2.

Now, we are going to derive its special cases. For *n* = 2 in (14)-(15),

(16) $$\begin{array}{c} r_{1}\left(t\right)=a_{0}+a_{1}\left(t-x\right). \end{array}$$

We find *a*_0_ and *a*_1_ such that

(17) $$\begin{array}{c} r_{1}\left(x\right)=f\left(x\right),\;\;r_{1}\left(w_{1}\right)=f\left(w_{1}\right). \end{array}$$

So, the two-point iterative scheme becomes

(18) w1=x+βfx,w2=x−fxfw1,x.

The iterative scheme (18) is the same as given by Steffensen [11] for *β* = 1; thus, is a particular case of our scheme given by (14)-(15).

For *n* = 3, we have

(19) $$\begin{array}{c} r_{2}\left(t\right)=\frac{a_{0}+a_{1}\left(t-x\right)}{1+b_{1}\left(t-x\right)}. \end{array}$$

We find *a*_0_, *a*_1_, and *b*_1_ using the following conditions:

(20) r2x=fx,  r2w1=fw1,r2w2=fw2.

By using conditions (20), we have

(21) a0=fx,a1=fw2,x+b1fw2,b1=fw1,x−fw2,xfw2−fw1.

Now, using (21), we have the following three-point iterative scheme:

(22) w1=x+βfx,w2=x−fxfw1,x,w3=x−fxfw2−fw1fw2fw1,x−fw1fw2,x.

For *n* = 4, we have the following rational interpolant:

(23) $$\begin{array}{c} r_{3}\left(t\right)=\frac{a_{0}+a_{1}\left(t-x\right)}{1+b_{1}\left(t-x\right)+b_{2}{\left(t-x\right)}^{2}}, \end{array}$$

such that

(24) r3x=fx,  r3w1=fw1,r3w2=fw2,  r3w3=fw3.

The conditions (24) are used to determine the unknowns *a*_0_, *a*_1_, *b*_1_, and *b*_2_. Thus, we attain a four-point iterative method as follows:

(25) w1=x+βfx,w2=x−fxfw1,x,w3=x−fxfw2−fw1fw2fw1,x−fw1fw2,x,w4=x−fxh1+h2+h3h1fw1,x+h2fw2,x+h3fw3,x,

where,

(26) h1=fw2fw3w3−w2,h2=fw1fw3w1−w3,h3=fw1fw2w2−w1.

For *n* = 5, we have the following five-point iterative scheme:

(27) w1x+βfx,w2=x−fxfw1,x,w3=x−fxfw2−fw1fw2fw1,x−fw1fw2,x,w4=x−fxh1+h2+h3h1fw1,x+h2fw2,x+h3fw3,x,w5=x−a0a1,

where,

(28) $$\begin{array}{c} r_{4}\left(t\right)=\frac{a_{0}+a_{1}\left(t-x\right)}{1+b_{1}\left(t-x\right)+b_{2}{\left(t-x\right)}^{2}+b_{3}{\left(t-x\right)}^{3}}, \end{array}$$

such that

(29) r4x=fx,  r4w1=fw1,r4w2=fw2,  r4w3=fw3,r4w4=fw4.

The interpolating conditions (29) yield

(30) a0=fx,a1=m1fw1,x+m2fw2,x+m3fw3,x   +m4fw4,xm1+m2+m3+m4−1,

where,

(31) m1=fw2fw3fw4   ×−w3−xw4−xw4−w3    +w2−xw4−xw4−w2   −w2−xw3−xh1fw4m2=fw1fw3fw4   ×w3−xw4−xw4−w3    −w1−xw4−xw4−w1   −w1−xw3−xh2fw4m3=fw1fw2fw4   ×−w2−xw4−xw4−w2    +w1−xw4−xw4−w1   −w1−xw2−xh3fw4m4=fw1fw2fw3   ×w2−xw3−xw3−w2    −w1−xw3−xw3−w1   +w1−xw2−xh3fw3,

and *h*_1_, *h*_2_, *h*_3_ are given as in (26). Hence, we obtain the following iterative method:

(32) w1=x+βfx,w2=x−fxfw1,x,w3=x−fxfw2−fw1fw2fw1,x−fw1fw2,x,w4=x−fxh1+h2+h3h1fw1,x+h2fw2,x+h3fw3,x,w5=x−fxm1+m2+m3+m4   ×m1fw1,x+m2fw2,x     +m3fw3,x+m4fw4,x−1.

We, now, give the convergence analysis of the proposed iterative methods (18), (22), (25), and (32).

## 3. Convergence Analysis

**Theorem 1 .**

Let us consider *ω*∈*I* as the simple root of sufficiently differentiable function *f* : *I*⊆*R* → *R* in the neighborhood of the root for interval *I*. If *x* is sufficiently close to *ω*, then, for every *β* ∈ *R*∖{−1}, the iterative methods defined by (18) and (22) are second and fourth order convergent, respectively, with the error equations given by

(33) en+1=c21+βen2+Oen3,en+1=c23−c2c3+2c23β+c23β2    −2c2c3β−c2c3β2en4+Oen5,

respectively, where,

(34) $$\begin{array}{c} c_{k}=\frac{1}{k!}\frac{f^{\left(k\right)}\left(\omega \right)}{f^{\prime }\left(\omega \right)},\;\;k=2,3,\ldots \end{array}$$

**Proof**

Let *x* = *ω* + *e*_*n*_, where *ω* is the root of *f* and *e*_*n*_ is the error at *n*th step. Now, using Taylor expansion of *f*(*x*) about the root *ω*, we have

(35) fx=f′ωen+c2en2+c3en3+c4en411111111+⋯+c8en8+Oen9,

where *c*_*k*_ is defined by (34). Taylor's expansions for *w*_1_ and *f*(*w*_1_) are

(36) w1=en+ω+βen+c2en2+c3en3      +c4en4+c5en5+Oen6,

(37) fw1 =f′ω1+βen+3βc2+c2+c2β2en21111111111+2c22β+2c22β2+c3+4βc31111111111111+3c3β2+c3β3en31111111111+5c2βc3+8c2β2c3+3c3β31111111111111+c4+5βc4+6β2c4+4c4β31111111111111+c4β4+c23β2en41111111111+c5+6βc5+10c5β2+10c5β3+5c5β4111111111111 +c5β5+6c2βc4+14c2c4β2+12c4c2β3111111111111+4c4c2β4+5c22c3β2+3c32β+6c32β2111111111111+3c3β3c22+3c32β3en5+Oen6.

Using (35), (36), and (37), we have

(38) w2=ω+c21+βen2  +−2c22−2c22β+2c3+3βc3+c3β2−c22β2en3  +4c23+5c23β−7c2c3−10c2βc3−7c2β2c3    +3c23β2−2c3β3c2+3c4+6βc4+4c4β2    +c4β3+c23β3en4+Oen5,

which shows that the method (18) is quadratically convergent for all *β* ∈ *R*∖{−1}. Again using Taylor expansion of *f*(*w*_2_), we have

(39) fw2=f′ωc21+βen21111111111111+−2c22−2c22β+2c31111111111111111+3βc3+c3β2−c22β2en31111111111111+5c23+7c23β−7c2c3−10c2βc31111111111111111−7c2β2c3+4c23β21111111111111111−2c3β3c2+3c4+6βc41111111111111111+4c4β2+c4β3+c23β3en4+Oen5.

Now, using (35)–(39), we see that the order of convergence of the method (22) is four and the error equation is given by

(40) en+1=c23+2c23β−c2c3−2c2c3β  −c2c3β2+c23β2en4+Oen5.

**Theorem 2 .**

Let us consider *ω*∈*I* as the simple root of sufficiently differentiable function *f* : *I*⊆*R* → *R* in the neighborhood of the root for interval *I*. If *x* is sufficiently close to *ω*, then for all *β* ∈ *R*∖{−1}, the iterative methods defined by (25) and (32) are eighth and sixteenth order convergent, respectively, with the error equations given by

(41) en+1=c22c25β4−3c3c23β4+4c25β3+c4c22β4      +2c32c2β4−12c3c23β3+6c25β2      −c4c3β4+4c4c22β3+8c32c2β3−18c3c23β2      +4c25β−4c4c3β3+6c4c22β2      +12c32c2β2−12c3c23β+c25−6c4c3β2      +4c4c22β+8βc2c32−3c23c3      −4βc3c4+c22c4+2c2c32−c3c4en8+Oen9,en+1=4c25c3c5−c32c4c5+2c2c32c42+2c2c33c5   −4c23c3c42−5c23c32c5−c24c4c5+18c24c32c4   −13c26c3c4−9c22c33c4+2c25c42+c34c4   −2c2c35−c27c5−7c29c3+3c28c4   +11c23c34−21c25c33+18c27c32   +2c22c3c4c5+c211c24β8  +⋯+4c25c3c5−c32c4c5+2c2c32c42+2c2c33c5      −4c23c3c42−5c23c32c5−c24c4c5      +18c24c32c4−13c26c3c4−9c22c33c4      +2c25c42+c34c4−2c2c35−c27c5−7c29c3      +3c28c4+11c23c34−21c25c33+18c27c32      +2c22c3c4c5+c211c24en16+Oen17,

where,

(42) $$\begin{array}{c} c_{k}=\frac{1}{k!}\frac{f^{\left(k\right)}\left(\omega \right)}{f^{\prime }\left(\omega \right)},\;\;k=2,3,\ldots \end{array}$$

**Proof**

Let *x* = *ω* + *e*_*n*_, where *ω* is the root of *f* and *e*_*n*_ is the error in the approximation at *n*th iteration. We will use (35), (37), (39), and (40) up to *O*(*e*_*n*_^30^) in this result and set

(43) w3=ω+c23+2c23β−c2c3−2c2c3β      −c2c3β2+c23β2en4   +4c3β3c22−2c24β3−c32β3−c4β3c2     −4c2β2c4+14c22β2c3−6c24β2−4c32β2     +18c22βc3−5c2βc4−8c24β−5c32β     −4c24−2c2c4+8c22c3−2c32en5   +⋯+Oen30.

Again, using Taylor expansion of *f*(*w*_3_), we have

(44) fw3=f′ωc23+2c23β−c2c3−2c2c3β111111111111111−c2c3β2+c23β2en411111111111111+4c3β3c22−2c24β3−c32β3−c4β3c211111111111111111−4c2β2c4+14c22β2c3−6c24β2−4c32β211111111111111111+18c22βc3−5c2βc4−8c24β−5c32β11111111111111111−4c24−2c2c4+8c22c3−2c32en511111111111111+⋯+Oen30.

Now, using (35)–(39), (43), and (44), we see that the method (25) has eighth order convergence with the error equation

(45) en+1=c22c25β4−3c3c23β4+4c25β3+c4c22β4     +2c32c2β4−12c3c23β3+6c25β2     −c4c3β4+4c4c22β3+8c32c2β3−18c3c23β2     +4c25β−4c4c3β3+6c4c22β2     +12c32c2β2−12c3c23β+c25−6c4c3β2     +4c4c22β+8βc2c32−3c23c3     −4βc3c4+c22c4+2c2c32−c3c4en8+Oen9.

To find the error equation of (32), we use (45) up to *O*(*e*_*n*_^30^) and set

(46) w4=c22c25β4−3c3c23β4+4c25β3+c4c22β4    +2c32c2β4−12c3c23β3+6c25β2−c4c3β4    +4c4c22β3+8c32c2β3−18c3c23β2    +4c25β−4c4c3β3+6c4c22β2    +12c32c2β2−12c3c23β+c25−6c4c3β2    +4c4c22β+8βc2c32−3c23c3    −4βc3c4+c22c4+2c2c32−c3c4en8   +⋯+Oen30.

Taylor expansion of *f*(*w*_4_) is

(47) fw4=f′ωc27β4+c24c4+c27+c4β4c24     −c22c4c3−c4c3β4c22+8c23c32β3     +2β4c32c23+8c23βc32+12c23β2c32     −18c25c3β2−12c25c3β−3β4c25c3     +6c24c4β2+4c24c4β3+4c24c4β     −12c25c3β3+6c27β2+4c27β3+4c27β     −3c25c3+2c23c32−6c3β2c4c22     −4c22c4βc3−4c4c22c3β3en8     +⋯+Oen30.

Hence, using (35)–(39), (43), (44), (46), and (47), we see that iterative method (32) is sixteenth order convergent with the error equation given by

(48) en+1=4c25c3c5−c32c4c5+2c2c32c42+2c2c33c511111111−4c23c3c42−5c23c32c5−c24c4c511111111+18c24c32c4−13c26c3c4−9c22c33c4+2c25c4211111111+c34c4−2c2c35−c27c5−7c29c311111111+3c28c4+11c23c34−21c25c33+18c27c3211111111+2c22c3c4c5+c211c24β81111111+⋯+4c25c3c5−c32c4c5+2c2c32c42+2c2c33c511111111111111−4c23c3c42−5c23c32c5−c24c4c5+18c24c32c411111111111111−13c26c3c4−9c22c33c4+2c25c42+c34c4−2c2c3511111111111111−c27c5−7c29c3+3c28c4+11c23c3411111111111111−21c25c33+18c27c32+2c22c3c4c511111111111111+c211c24en16+Oen17.

**Remark 3 .**

From Theorems 1 and 2, it can be seen that the iterative schemes (18), (22), (25), and (32) are second, fourth, eighth, and sixteenth order convergent requiring two, three, four, and five functional evaluations, respectively. Hence, the proposed iterative schemes (18), (22), (25), and (32) are optimal in the sense of the hypothesis of Kung and Traub [9] with the efficiency indices 1.414, 1.587, 1.681, 1.741. Also, it is clear that (14)-(15) is a general *n*-point scheme with optimal order of convergence 2^*n*−1^. The efficiency index of this scheme is 2^(*n*−1)/*n*^.

## 4. Numerical Results

In this section, we present some test functions to demonstrate the performance of the newly developed sixteenth order scheme (32) (FNMS-16). For the sake of comparison, we consider the existing higher order convergent methods based on rational interpolation. We consider the fourteenth order method of Soleymani and Sharifi (4) (SS-14), the sixteenth order method of Soleymani et al.(7) (SSS-16), and the sixteenth order method of Sharma et al.(9) (SGG-16). All the computations for the above-mentioned methods are performed using Maple 16 with 4000 decimal digits precision. The test functions given in Table 1 are taken from [1, 2, 10]. We used almost all types of nonlinear functions, polynomials, and transcendental functions to test the new methods.  Table 2 shows that the newly developed sixteenth order methods are comparable with the existing methods of this domain in terms of significant digits and number of function evaluations per iteration. In many examples, the newly developed methods perform better than the existing methods. It can also be seen from the tables that, for the choice of initial guess, near to the exact root or far from the exact root, the performance of the new methods is better.

## 5. Attraction Basins

Let *ω*_*i*_ be the roots of the complex polynomial *p*_*n*_(*x*), *n* ≥ 1, *x* ∈ *C*, where *i* = 1,2, 3,…, *n*. We use two different techniques to generate basins of attraction on MATLAB software. We take a square box of [−2,2] × [−2,2] ∈ *C* in the first technique. For every initial guess *x*_0_, a specific color is assigned according to the exact root, and dark blue is assigned for the divergence of the method. We use |*f*(*x*_*k*_)| < 10^−5^ as the stopping criteria for convergence and the maximum number of iterations are 30. “Jet” is chosen as the colormap here. For the second technique, the same scale is taken but each initial guess is assigned a color depending upon the number of iterations for the method to converge to any of the roots of the given function. We use 25 as the maximum number of iterations; the stopping criteria are the same as above and colormap is selected as “hot.” The method is considered divergent for that initial guess if it does not converge in the maximum number of iterations and this case is displayed by black color.

We take three test examples to obtain basins of attraction, which are given as *p*_3_(*x*) = *x* ^3^ − 1,  *p*_4_(*x*) = *x* ^4^ − 10*x* ^2^ + 9, and *p*_5_(*x*) = *x* ^5^ − 1. The roots of *p*_3_(*x*) are 1.0, −0.5000 + 0.86605*I*, and −0.5000 − 0.86605*I*, the roots of *p*_4_(*x*) are −3, 3, −1, 1, and for *p*_5_(*x*) roots are 1.0, 0.3090 + 0.95105*I*, −0.8090 + 0.58778*I*, −0.8090 − 0.58778*I*, and 0.30902 − 0.95105*I*.

**Figure 1:**
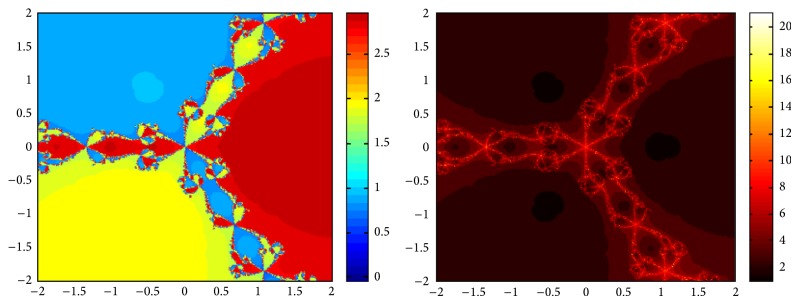
Basins of attraction of method (7) for *p*_3_(*x*).

**Figure 2:**
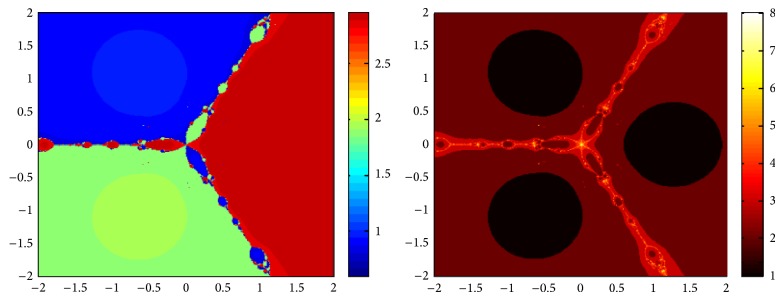
Basins of attraction of method (9) for *p*_3_(*x*).

**Figure 3:**
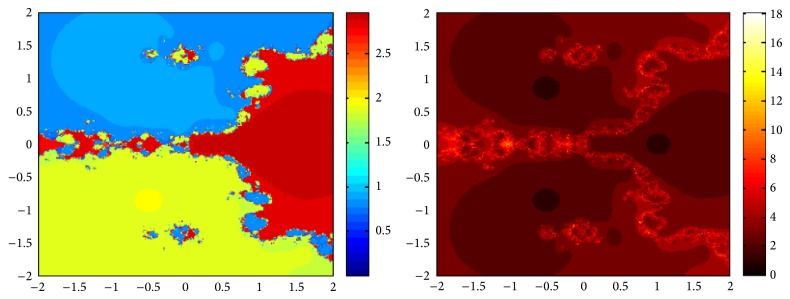
Basins of attraction of method (32) for *p*_3_(*x*).

**Figure 4:**
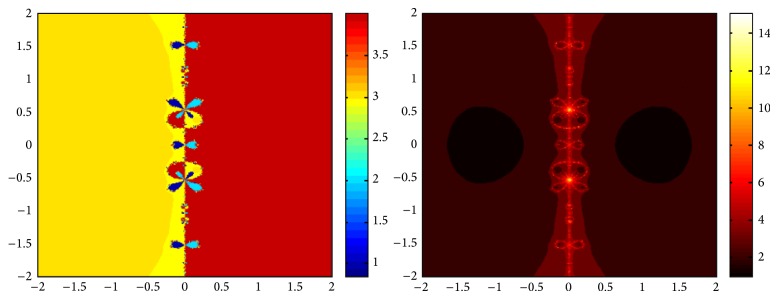
Basins of attraction of method (7) for *p*_4_(*x*).

**Figure 5:**
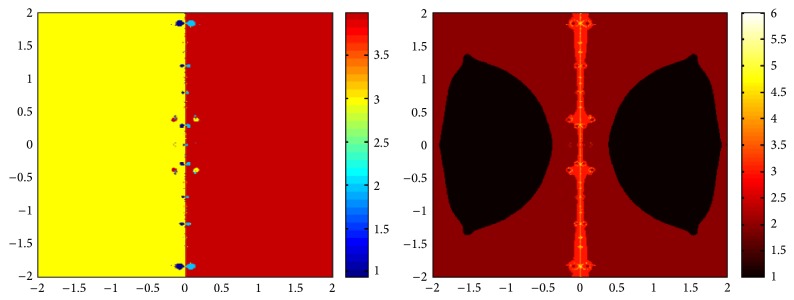
Basins of attraction of method (9) for *p*_4_(*x*).

**Figure 6:**
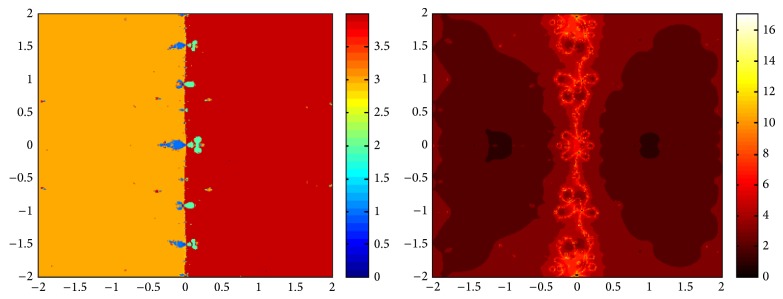
Basins of attraction of method (32) for *p*_4_(*x*).

**Figure 7:**
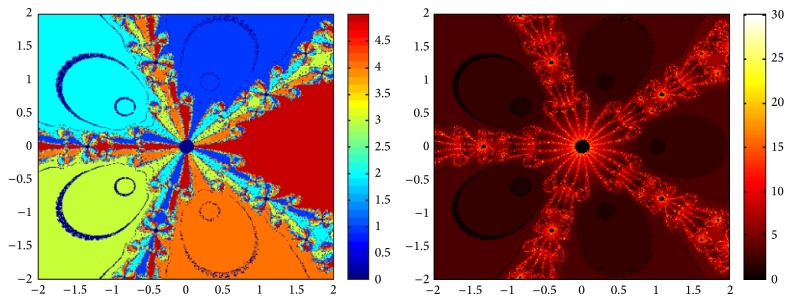
Basins of attraction of method (7) for *p*_5_(*x*).

**Figure 8:**
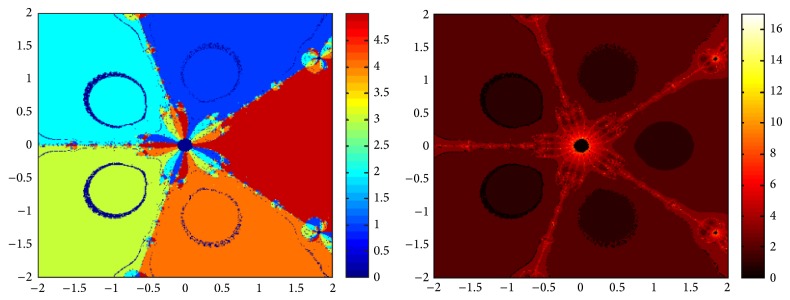
Basins of attraction of (9) for *p*_5_(*x*).

**Figure 9:**
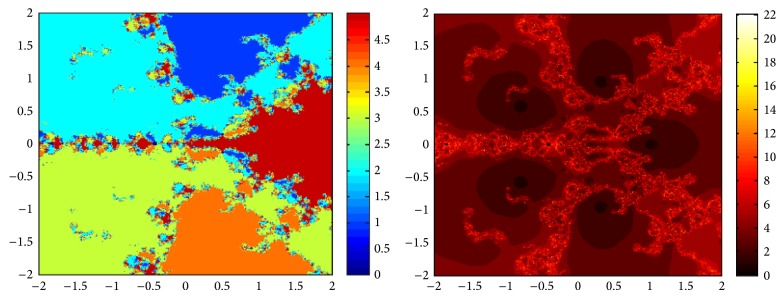
Basins of attraction of (32) for *p*_5_(*x*).

We compare the results of our newly constructed method (32) with some existing methods (7) and (9), as given in Section 1. Figures 1, 2, 3, 4, 5, 6, 7, 8, and 9 show the dynamics of the methods (7), (9), and (32) for the polynomials *x* ^3^ − 1, *x* ^4^ − 1, and *x* ^5^ − 1. Two types of attraction basins are given in all figures. One can easily see that the appearance of darker region shows that the method consumes a fewer number of iterations. Color maps for both types are given with each figure which shows the root to which an initial guess converges and the number of iterations in which the convergence occurs.

## Figures and Tables

**Table 1:** Test functions and exact roots.

Numerical example	Exact roots
$f_{1}(x)=\sqrt{x^{4}+8}\sin(\pi /(x^{2}+2))+x^{3}/(x^{4}+1)-\sqrt{6}+8/17$	*ω*_1_ = −2
*f*_2_(*x*) = sin⁡⁡(*x*) − *x*/100	*ω*_2_ = 0
*f*_3_(*x*) = (1/3)*x* ^4^ − *x* ^2^ − (1/3)*x* + 1	*ω*_3_ = 1
*f*_4_(*x*) = *e* ^sin⁡⁡(*x*)^ − 1 − *x*/5	*ω*_4_ = 0
*f*_5_(*x*) = *xe* ^*x*^2^^ − (sin⁡⁡(*x*))^2^ + 3cos⁡(*x*) + 5	*ω*_5_ ≈ −1.207647827130919
*f*_6_(*x*) = *e* ^−*x*^ + cos⁡(*x*)	*ω*_6_ ≈ 1.746139530408013
*f*_7_(*x*) = 10*xe* ^−*x*^2^^ − 1	*ω*_7_ ≈ 1.679630610428450
*f*_8_(*x*) = *x* ^3^ + 4*x* ^2^ − 15	*ω*_8_ ≈ 1.631980805566063

**Table 2:** Comparison of various iterative methods.

*f*(*x*), *x*_0_	(SS-14)	(SGG-16)	(SSS-16)	(FNMS-16)
*f*_1_, *x*_0_ = −1.2				
|*f*_1_(*x*_1_)|	.3*e* − 13	.1*e* − 15	.1*e* − 13	.1*e* − 15
|*f*_1_(*x*_2_)|	.1*e* − 181	.6*e* − 248	.1*e* − 211	.5*e* − 245
|*f*_1_(*x*_3_)|	.6*e* − 2538	.6*e* − 3751	.7*e* − 3379	.6*e* − 3916
*f*_1_, *x*_0_ = −3				
|*f*_1_(*x*_1_)|	.1*e* − 5	.3*e* − 6	.3*e* − 7	.2*e* − 8
|*f*_1_(*x*_2_)|	.4*e* − 75	.1*e* − 96	.2*e* − 113	.1*e* − 132
|*f*_1_(*x*_3_)|	.1*e* − 1050	.3*e* − 1545	.2*e* − 1812	.5*e* − 2123
*f*_2_, *x*_0_ = 1.5				
|*f*_2_(*x*_1_)|	.95	.95	.64	.3*e* − 4
|*f*_2_(*x*_2_)|	.1*e* − 10	.1*e* − 4	.3*e* − 6	.5*e* − 100
|*f*_2_(*x*_3_)|	.2*e* − 88	.5*e* − 90	.1*e* − 111	.4*e* − 1632
*f*_2_, *x*_0_ = 3				
|*f*_2_(*x*_1_)|	.2*e* − 24	.1*e* − 25	.3*e* − 27	.2*e* − 44
|*f*_2_(*x*_2_)|	.8*e* − 358	.8*e* − 425	.2*e* − 452	.9*e* − 743
|*f*_2_(*x*_3_)|	0	0	0	0
*f*_3_, *x*_0_ = 0.5				
|*f*_3_(*x*_1_)|	.5*e* − 8	.6*e* − 9	.3*e* − 9	.9*e* − 10
|*f*_3_(*x*_2_)|	.8*e* − 114	.3*e* − 144	.4*e* − 149	.6*e* − 238
|*f*_3_(*x*_3_)|	.2*e* − 1595	.1*e* − 2307	.1*e* − 2386	0
*f*_3_, *x*_0_ = 1.5				
|*f*_3_(*x*_1_)|	.2*e* − 11	.2*e* − 13	.2*e* − 11	.1*e* − 11
|*f*_3_(*x*_2_)|	.2*e* − 158	.6*e* − 214	.1*e* − 179	.6*e* − 183
|*f*_3_(*x*_3_)|	.3*e* − 2218	.8*e* − 3423	.2*e* − 2869	.6*e* − 2935
*f*_4_, *x*_0_ = 5				
|*f*_4_(*x*_1_)|	.8*e* − 1	.25	.8*e* − 1	.1*e* − 2
|*f*_4_(*x*_2_)|	.4*e* − 15	.1*e* − 6	.3*e* − 16	.3*e* − 56
|*f*_4_(*x*_3_)|	.1*e* − 213	.7*e* − 109	.9*e* − 262	.1*e* − 914
*f*_4_, *x*_0_ = 4				
|*f*_4_(*x*_1_)|	.92	.9*e* − 1	2.51	.1*e* − 17
|*f*_4_(*x*_2_)|	.4*e* − 5	.1*e* − 25	.6*e* − 3	.3*e* − 295
|*f*_4_(*x*_3_)|	.1*e* − 73	.1*e* − 423	.6*e* − 49	0
*f*_5_, *x*_0_ = −1				
|*f*_5_(*x*_1_)|	.1*e* − 3	.1*e* − 7	.8*e* − 4	.7*e* − 5
|*f*_5_(*x*_2_)|	.2*e* − 66	.5*e* − 144	.9*e* − 80	.4*e* − 92
|*f*_5_(*x*_3_)|	.4*e* − 946	.8*e* − 2327	.1*e* − 1294	.6*e* − 1488
*f*_5_, *x*_0_ = −0.6				
|*f*_5_(*x*_1_)|	.2*e*44770	.4*e* − 1	.2*e*44770	.1
|*f*_5_(*x*_2_)|	.4*e*44769	.4*e* − 40	.7*e*44768	.3*e* − 23
|*f*_5_(*x*_3_)|	.5*e*44767	.4*e* − 664	.2*e*44767	.8*e* − 386
*f*_6_, *x*_0_ = 0.5				
|*f*_6_(*x*_1_)|	.6*e* − 7	.9*e* − 9	.3*e* − 8	.1*e* − 14
|*f*_6_(*x*_2_)|	.8*e* − 116	.1*e* − 152	.7*e* − 145	.1*e* − 254
|*f*_6_(*x*_3_)|	.1*e* − 1748	.7*e* − 2456	.9*e* − 2332	.1*e* − 3999
*f*_6_, *x*_0_ = 3				
|*f*_6_(*x*_1_)|	.7	.53	.79	0.7*e* − 8
|*f*_6_(*x*_2_)|	.9*e* − 5	.4*e* − 16	.2*e* − 5	.2*e* − 145
|*f*_6_(*x*_3_)|	.7*e* − 102	.1*e* − 269	.5*e* − 125	0.3*e* − 2345
*f*_7_, *x*_0_ = 0				
|*f*_7_(*x*_1_)|	.1*e* − 6	.2*e* − 9	.1*e* − 5	.8*e* − 12
|*f*_7_(*x*_2_)|	.5*e* − 99	.3*e* − 161	.6*e* − 98	.1*e* − 199
|*f*_7_(*x*_3_)|	.3*e* − 1394	.6*e* − 2562	.5*e* − 1575	.3*e* − 3205
*f*_7_, *x*_0_ = 2.2				
|*f*_7_(*x*_1_)|	1	.9*e* − 2	1	.8*e* − 8
|*f*_7_(*x*_2_)|	1	.6*e* − 40	D	.9*e* − 136
|*f*_7_(*x*_3_)|	D	.5*e* − 651	D	.4*e* − 2183
*f*_8_, *x*_0_ = 0.5				
|*f*_8_(*x*_1_)|	.1*e*7	.97	.1*e*7	1.0
|*f*_8_(*x*_2_)|	16856.81	.2*e* − 25	16856.81	.8*e* − 15
|*f*_8_(*x*_3_)|	183.46	.3*e* − 435	183.46	.1*e* − 256
*f*_8_, *x*_0_ = 1				
|*f*_8_(*x*_1_)|	.2*e* − 2	.5*e* − 5	.2*e* − 2	.6*e* − 3
|*f*_8_(*x*_2_)|	.2*e* − 65	.1*e* − 109	.2*e* − 65	.7*e* − 67
|*f*_8_(*x*_3_)|	.6*e* − 1073	.1*e* − 1782	.6*e* − 1073	.1*e* − 1089

^*^D stands for divergence.
